# Challenges in recognizing and discussing changes in a resident’s condition in the palliative phase: focus group discussions with nursing staff working in nursing homes about their experiences

**DOI:** 10.1186/s12904-024-01479-3

**Published:** 2024-06-10

**Authors:** C. Bagchus, M. S. Zee, J. T van der Steen, M. S. Klapwijk, N. Lemos Dekker, B. D. Onwuteaka-Philipsen, H. R. W. Pasman

**Affiliations:** 1 Department of Public and Occupational Health, Expertise Center for Palliative Care, Amsterdam UMC, VU University, Amsterdam, the Netherlands; 2 Faculty of Health, University of Applied Sciences Leiden, Leiden, the Netherlands; 3 Department of Public Health and Primary Care, Leiden University Medical Center, Hippocratespad 21, Gebouw 3, P.O. Box 9600, 2300 RC Leiden, The Netherlands; 4 Department of Primary and Community Care and Radboudumc Alzheimer center, Radboud university medical center, P.O. Box 9600, 6500 HB Nijmegen, The Netherlands; 5 Institute of Cultural Anthropology and Development Sociology, Leiden University, Leiden, the Netherlands

**Keywords:** Nursing homes, Palliative care, Terminal care, Nurses

## Abstract

**Background:**

Most nursing home residents have complex care needs, require palliative care and eventually die in these facilities. Timely recognition of changes in a resident’s condition is crucial for providing appropriate care. Observations by nursing staff play a significant role in identifying and interpreting these changes.

**Methods:**

Focus group discussions were conducted with nursing staff from ten nursing homes in the Netherlands to explore their experiences and challenges in recognizing and discussing changes in a resident’s condition. These discussions were analysed following the principles of thematic analysis.

**Results:**

The analysis of the challenges nursing staff face in identifying and interpreting changes in a resident’s condition, resulted in three themes. First, that recognizing changes is considered complex, because it requires specialized knowledge and skills that is generally not part of their education and must partly be learned in practice. This also depends on how familiar the nursing staff is with the resident. Furthermore, different people observe residents through different lenses, depending on their relation and experiences with residents. This could lead to disagreements about the resident’s condition. Lastly, organizational structures such as the resources available to document and discuss a resident’s condition and the hierarchy between nursing home professionals often hindered discussions and sharing observations.

**Conclusion:**

Nursing staff’s experiences highlight the complexity of recognizing and discussing changes in nursing home residents’ conditions. While supporting the observational skills of nursing staff is important, it is not enough to improve the quality of care for nursing home residents with palliative care needs. As nursing staff experiences challenges at different, interrelated levels, improving the process of recognizing and discussing changes in nursing home residents requires an integrated approach in which the organization strengthens the position of nursing staff. It is important that their observations become a valued and integrated and part of nursing home care.

**Supplementary Information:**

The online version contains supplementary material available at 10.1186/s12904-024-01479-3.

## Background

Residential and nursing home care is intended for people with highly complex care needs who need 24/7 care [1]. In practice, the vast majority of nursing home residents has serious physical limitations, multiple chronic diseases and many have cognitive impairments or dementia [2]. Most nursing home residents already have palliative care needs at the time of admission and for most long-term residents the nursing home will eventually be the place of their death [3, 4]. Given these facts, it can be stated that admission to a nursing home marks such a high vulnerability that most residents would benefit from a palliative approach from the time of admission to the nursing home.

Residents’ physical condition and functional status will naturally change over time, but also residents’ social, emotional and spiritual needs will change and have to be considered to provide appropriate care [5]. Therefore, it is important that changes in a resident’s condition are recognized in time to assure timely adjustment of daily care routines and care plans, and to timely recognise the dying phase.

Many residents have problems recognizing and understanding changes in their own condition and to communicate how they feel and what they need. If this is the case, based on observing residents, *others* must interpret the meaning of the observed changes in terms of needs. Subsequently, these *others* must discuss their observations and interpretations to decide together how the needs of the residents can be best met [6]. Therefore, timely and precise observations of verbal and non-verbal behaviour by caregivers are of major importance in the process of recognizing changes.

In the context of nursing homes, palliative care relies to a large extent on the observations of nursing staff [7]. As nursing staff (especially care assistants, and nursing aids) spend most of their time in direct contact with residents, they might be the first to recognize changes. During daily care, nursing staff might observe pain and other forms of discomfort, but also restlessness, sadness or fear.

However, in practice, timely identification of changing palliative care needs can be challenging, especially in people with dementia [8–11]. Nursing staff might not be fully aware of the importance of their role [12]. Additionally, the complexity of the constantly changing palliative care needs requires such specialized knowledge and skills that nursing staff is not always properly equipped to provide palliative care [10, 13–15]. When changes are not recognized and addressed timely, comfort and well-being are at risk [16, 17] and death can be unexpected [18]. Furthermore, observations tend to focus on physical care needs, bringing the risk of missing of social, emotional and spiritual needs [19].

Observations of nursing staff are important. Supporting their observational skills is likely to improve the quality of care for vulnerable nursing home residents. However, little is known about the daily experiences of nursing staff with recognizing changing care needs of nursing home residents. Their experiences may contain valuable information of the underlying challenges nursing staff faces in their daily work. It also might provide clues on how to support nursing staff with their pivotal role in palliative care for nursing home residents. None of the above mentioned studies [8–12] specifically looked into what exactly makes this first essential step, i.e. signalling changes and needs, in providing appropriate care to nursing home patients difficult. Therefore, the aim of this study is to better understand the challenges in the process of signalling and discussing changes in the condition of nursing home residents from the perspective of nursing staff working in nursing homes.

## Methods

### Design

Semi-structured focus group discussions with nursing staff teams of 10 Dutch nursing homes, using an open, inductive (constructivist) approach. These focus group discussions were a first step in a larger action research project (the SigMa project) and are analysed separately for the current paper.

### Dutch setting

Dutch nursing home care shows a substantial variability in nursing home stay, but the people with dementia still have a median stay of approximately 2 years [20, 21]. Dutch nursing homes have specialized units tailored for people with dementia or chronic illnesses, providing care until the end of life. The nursing staff in these facilities comprises individuals with varying levels of education, including certified nursing assistants, nursing aids and registered nurses. Due to staffing shortages, the presence of temporary staff is often necessary, although precise figures on their frequency are lacking. Medical care in Dutch nursing homes is typically provided by elderly care physicians who are on staff, often in collaboration with nurses practitioners or physicians in training [22]. There can be significant differences in on-site presence of nurses and physicians [23], which can impact the ability to recognize changes in residents’ condition, as daily care is provided by nursing assistants and nursing aids with less training in symptom assessment and relieve.

### Participants

Participants were nurses, nurse assistants and nursing aids from ten nursing staff teams of 10 nursing homes. Selection of nursing homes and teams was done for the larger action research study, for which nursing homes could apply. For the action research it was important that teams were fairly stable teams in terms of team culture, management and staff turnover, and were not too busy with other projects, training programs or developments in order to manage the workload of the teams. Furthermore, the teams had to be open to change and motivated to improve palliative care. Recruitment for the focus groups was done via the team manager aiming for a representation of the team with respect to educational level, experiences of and knowledge about palliative care. The managers themselves did not participate in the focus group discussion. Nursing homes were located in different regions of the Western part of the Netherlands and located in big cities as well as small villages. They differed in size and composition (only psychogeriatric care, and combined somatic and psychogeriatric care).

### Data collection

The focus group discussions (*N* = 10) this paper reports on took place at the start of the SigMa-project (September – December 2017). Focus group discussions with 5 to7 participants were planned on location, and lasted between 1,5 and 2 h with a break in between. The first focus group discussion was facilitated by CB and NLD (both female anthropologists and post-doctoral researchers) together, the other focus group discussions were facilitated by either CB or NLD. To guide the focus group discussions, a topic list (Appendix 1) was used, including the meaning of palliative care according to the participants, signs of change and gut feelings, current procedures regarding recognizing and discussing changes, roles and responsibilities of different staff members, the role of residents and relatives, challenges and support needs. In the focus groups, participants were asked to share examples of cases of good, timely adjustment of care and cases of care lagging behind the changing needs of a resident.

### Data analysis

All focus group discussions were recorded and transcribed verbatim. After transcription, the data were anonymized. From the first focus group discussion onward, the experiences shared in the focus group discussions were discussed in the project group. Subsequently, the findings were discussed with the participants of the focus group to verify if our interpretation of the data was accurate. We analysed the data using MAXQDA software to support analyses of qualitative data, following the principles and six steps of thematic analysis ( [24]. We started with a thorough re-reading of the transcripts (CB, MZ), followed by open coding of relevant information regarding the aim of this study (CB, MZ). Thereafter, open codes were sorted in different subcodes, codes and overarching themes (CB, MZ). (see appendix 2 for coding scheme). This process was discussed with the authors of this paper (i.e. the project group) at different moments, to refine the themes and avoid bias. We feel that data saturation is reached, since similar challenges appeared in the different focus group discussions and in the later focus group discussions no new challenges came up.

### Ethics

All staff members who participated in the focus group discussions were informed about the research and signed for informed consent. The Medical Ethics Committee of Leiden University Medical Center, the Netherlands, declared that the study was exempt from the Medical Research Involving Human Subjects Act (P17.256).

## Results

Participants (i.e. nursing staff in the focus groups) defined good palliative care as care focussed on comfort, taking into account someone’s personal needs and preferences. In their examples of good palliative care, they referred to situations in which residents felt comfortable and care plans were pro-actively adjusted to prevent any discomfort. They emphasized that these were often situations in which relatives, nursing staff and physicians agreed on what was best for the resident. Participants explained that consensus on the desired directions of care makes it possible to act fast when a resident is no longer comfortable. Related to this, they shared examples of situations in which they took or were given extra time to care for residents who needed extra attention. Participants explained that taking this extra time is easier to realize when there is agreement on a resident’s needs.

In all focus group discussions, it was acknowledged that timely recognition of changes in a resident’s condition is essential to quality of palliative care. Participants provided examples illustrating how quality of care was negatively affected when changes in a resident’s conditions were not recognised at all, or not recognised in time: *“I do think that we often act too late. What I just said, at this location we have a resident that is actually in the terminal phase. But we don’t act on it! Actually, that’s very strange… “ (*participant FG 1) and Some case keep me awake at night. When a resident can no longer eat. And stilI I have to give her all that bullshit [medication].

And even when changes are recognized, timely adjustment of care plans is not evident: *“When we identify a problem and report this to the elderly care physician, the psychologist or the physiotherapist, only then they come in action. And that can take some time… In the meantime we see the situation of resident getting worse. “ (participant FG 4).*

Our analysis of the challenges participants described in their everyday work, resulted in three themes: (1) recognising changes is complex, (2) different stakeholders look at the resident through different lenses and (3) organisational structures can hinder discussions on observations. See appendix 2 for a coding scheme with themes, codes and subcodes.

### Theme 1: Recognising changes is complex

Overall, participants mentioned that recognizing changes in a resident’s condition is complex. Care assistants and nurse aids found that their professional training included too little training in palliative care and the curriculum was mostly restricted to care in the last days of life. Recognizing changes before the terminal phase was not taught, but learned on the job. Based on their own experiences, experiences of colleagues and in some cases additional training, these participants developed a *“*gut feeling”, a highly individual, intuitive way of recognizing that “something” is different: *“Sometimes I can’t put my finger on it, but it just doesn’t feel right, I just see things that are not as they normally are…”*(participant FG 1). Trying to make their “gut feeling” more explicit, they explained that this feeling is usually based on subtle signs of overall deterioration that will only be recognized by people who are familiar with the resident, for example, a resident seemed more tired, has a different look in her r eyes, smiles less often, swears more often or is more demanding towards nursing staff: *Some cases keep me awake at night. When a resident can no longer eat. And still I have to give her all that bullshit [medication]”* (participant FG 7).

To notice these subtle changes, participants mentioned that they rely on their knowledge as to what is normal for this person: *“It always surprises me that I can feel my residents, especially when you are close them. Even in advanced dementia. I know what makes them feel good, what they like.”* (participant FG 1).

Some participants had experienced that these signals are missed by temporary staff who do not know the resident well and therefore, miss a reference point to compare their observations.

On the other hand, participants mentioned that being too involved might blur their ability to make objective observations. A complicating factor is that many residents are limited in their ability to communicate how they feel, what they need or what they want. Also, in relation to this, participants emphasized the importance of knowing a resident. To understand the impact of observed changes for the resident, it is necessary to know the resident personally, know their preferences and normal physical appearance: ”*As a nursing aid, you know your resident. I mean, the elderly care physician may have more [medical] knowledge, but you know your resident and you know how he reacts and what is important to him.”* (participant FG 1).

Recognizing changes is further complicated by the nature of the changes in the condition of nursing home residents. Especially participants who took care of people with dementia experienced that subtle signs of deterioration are difficult to identify. In contrast to major events that mark deterioration such as a broken hip, pneumonia or cerebral infarction, an accumulation of subtle changes–that together possibly make up a major change–is easily missed. *“There are people we care for in the same way every day, for years. That is just the way it goes. And of course their physical health is changing, but the changes are subtle, hard to observe, And at some point they end up in lying in bed and then you realize you missed the signs.”* (participant FG 1).

On the other hand, participants expressed that the palliative and terminal phase can be very unpredictable *“Signals can be ambiguously. Sometimes a resident recovers unexpectedly. How to deal with that? Indeed, that is an important point. – Yes! That is why this is so complex”. ”(participants* FG 6). Conditions can improve or worsen unexpectedly. Even in the context of constant deterioration, a death of the resident might still feel unexpected.

### Theme 2: Different people observe residents through different lenses

A second theme that came up was that participants often encountered people who look in a different way at a resident: *“I’m thinking about that resident, he’s sleeping in his chair all afternoon. When we would leave him in bed, he would rest better, he would be much more comfortable, he would not get restless… - I agree, there are people who can indeed better stay in bed… But there are also people who insist on getting these residents out of bed, at any price.“* (participants FG 4).

Perspectives could differ between and among colleagues, on-staff physicians and relatives. Participants experienced that even within the team, colleagues observed different things and have different thoughts on what is best for a resident. *“We have a lot of miscommunication. One observes something, the other doesn’t”* (participant FG 10). Especially when it comes to pain and other forms of discomfort, disagreements are common, for instance about whether a resident is comfortable spending time in the shared living room or not. Participants mentioned that although all staff members strive for comfort, they might disagree on a resident being comfortable or not, and on how comfort has to be realized. They gave various explanations for these differences such as the individual character of their “gut feeling”, the personal relationship staff members have with residents that influence their interpretations, but also that observations depend on the shift a staff member most commonly works, as some changes are only observed during specific times of the day such as restlessness or fear at night or difficulty getting up in the morning.

Additionally, participants shared that they are regularly confronted with relatives with different views on a resident’s condition. Moreover, they found that in general, relatives have a different view on the resident’s condition than nursing staff has, for example on overall prognosis, but also on physical and cognitive abilities, energy level or the number of stimuli someone can handle. Participants noticed that relatives typically see a resident during their best time of the day. They are not around when the resident wakes up or later during the day, when residents become restless and agitated. Another explanation they gave, is that relatives find it hard to accept the deteriorating health and the nearing death of their loved one: *“The family is also important, that’s challenging sometimes. – The family doesn’t want to see that the resident is in the last phase of life. – Their understanding dementia; knowledge is lacking. – Yes! And acceptance as well.“* (participants FG 2).

Participants expressed that some relatives do see the subtle signs of deterioration, but their emotions hinder them to accept the implications: even when relatives see that someone is exhausted, they don’t want them to spend more time in bed during the day.

Participants also mentioned that their views sometimes differ from the views of the physicians involved. They explained that under normal circumstances, the physician does not see the residents regularly, while nursing staff observes the resident during entire shifts, generally multiple days a week. Therefore, nursing staff has more time to observe and are more familiar with the residents, so divergent behaviour will be noticed faster. Furthermore, participants shared their experience that elderly care physicians observe a resident mainly from a medical perspective and prefer objective measurements that do not necessarily match the more subjective “gut feeling” of the nursing staff. Another explanation participants gave for the different observations of the elderly care physicians is that some residents pretend to feel better when the physician visits, than they actually do: *“Our residents are from the generation that when the doctor or the pastor comes to visit, they sit up straight for a moment and pretend to be alright. And they are exhausted afterwards. The others don’t see that.”* (participant FG 1).

Together, these complementary perspectives form a good representation of the resident’s condition (and therefore additional needs), since residents cannot always express their needs themselves. However, participants said that when there is no agreement with the team or with the elderly care physician on what is best for a resident, the tendency is to wait and see. They gave numerous examples of situations in which staff members believed that care plans did not fit the functioning of a residents, that medical examinations were unnecessary, that residents were overestimated by relatives, or that residents were exhausted because the daily routines did not fit their energy levels anymore. This frustrated nursing staff because they felt not taken seriously and felt powerless because they could not do what they thought was best for a resident: “*It is frustrating, that others do not rely on what we observe, what we recommend. – Yes. Yes, just what she says. We simply observe things, have experience working with a resident. It [discussing what is best for a resident] sometimes becomes such a tug-of-war…”* (participants FG 4).

### Theme 3: Organisational structures can hinder discussions on observations

The challenge of getting on the same page with colleagues, relatives and elderly care physicians was related to the communication structures within the organization. Participants stated that the electronic health record used within the organization, does not accommodate the documentation of subtle or subjective observations. As the electronic health record in theory is the most appropriate way to share observations with others, information that does not fit the system risks getting lost. *“Yes, but how much freedom do you have in that [electronic health record]? I feel limited, I’m stuck in a certain routine. While sometimes I feel like writing: Well, I don’t know, I might be crazy, but that lady did this and I don’t think it’s normal, I just can’t put my finger on it”* (participant FG 7). Additionally, across all focus group discussions, participants shared the experience that even well-documented information is being missed because in the hectic of daily practice, time for thorough reading of the reports is limited.

Additionally, participants said that sharing and discussing observations with colleagues or physicians face-to-face can be challenging. Time to sit down and discuss observations with others, such as the observations that do not fit in the electronic health record, is scarce. Time to discuss the day at shift changes has been abolished in many organizations, and time to discuss during shifts can be hard to find: *“I think there is an underlying problem, the structure of our shifts. I think a little more overlap between our shifts would help. Now, we actually work alone most of the time. – Yes, we work so soloistic, it’s challenging to simple provide care are a team, to get all on the same page.”* (participants FG 1).

They also stated that while there are recurrent multidisciplinary meetings to establish care plans with the elderly care physician and relatives, the frequency of these meetings does not always match the speed of the changing condition. In the meantime, nursing staff has to arrange ad hoc meetings with relatives and the elderly care physicians to discuss whether a change in care plans or approach of a resident is needed. Since most of the time, there is not an elderly care physician present 24/7, observations are discussed remotely or physicians should come and visit.

Finally, hierarchy within the organisations was seen as a hindering factor as participants felt their opinions were not taken seriously by professionals higher on the hierarchal ladder. *“We all know that we all have a different level of education. When we say something, you can take that seriously. We are not stupid. We are trained to do this work.”* (participant FG 10) This was especially the case for attending physicians during the nights and weekends whom they did not know well:

*“The physicians still seems to feel reluctance. Where does that come from? Do they have no confidence? Don’t they trust in our knowledge and skills?”* (participant FG 1).

Participants were familiar with the feeling of having to fight to be heard by physicians who did not know them or the residents well. Some participants added that relatives value the physician’s opinion more than the nursing staff’s opinions, although they spend way more time with the resident. Participants explained that this is less hindering when the people around the residents know each other and trust one another, implying that then timely adjustment of care was more likely.

## Discussion

This research shows that recognizing and discussing changes in the condition of nursing home residents is a complex process characterized by challenges on different levels. On an individual level, the experiences of nursing staff indicate that recognizing changes is highly complex as it requires specialized knowledge and skills. This is generally not part of their education and above that it cannot all be learned in professional training, but must be partly learned in practice and can only reach its full potential when a staff member knows a resident well. In collaboration with other professionals, residents and relatives, nursing staff are confronted with people who observe residents through different lenses and have different perspectives on what is best for a resident. Finally, nursing staff experience that organizational structures can be a hindering factor in bringing the different perspective together.

Poor palliative care training of nursing home staff still is a hindering factor, as is also found in other countries [15, 25, 26]. In particular nursing aids and care assistants, the people who are most likely to observe changing care needs first-hand and are responsible for providing palliative care, lack professional training. More attention for palliative care training of nursing home staff might improve their ability to provide the best palliative care [10, 15, 25, 26], although Fryer et al. found that lower educated staff preferred hands-on education delivered by peers, rather than formal education [27].

However, education on palliative care is just one element of what nursing staff needs to recognize changing needs. Experiential knowledge and personal knowledge of the individual resident is at least as important, although more intuitive and thereby harder to make explicit and to communicate. More acknowledgement of the value of the unique knowledge and the pivotal role of nursing staff is needed to make nursing staff feel valued and taken seriously [19, 27, 28]. Furthermore, it is important to make optimal use of this unique knowledge to provide palliative care for people with constantly, subtle changing needs. Therefore, we suggest to carefully listen to the insights shared by people who spend a lot of time with residents on a regular basis, including living room assistants, nutrition assistants and volunteers, since each brings their own unique perspective. This is likely to be especially important in this time of staff shortages, in which more temporary staff will take care of residents, as participants mentioned that you have to know the resident very well to recognize changes in their condition.

Our study underlines the importance of personal relations in the process of recognizing and discussing changing needs in nursing home residents in the palliative phase. This is in line with research of others who found that staff must be familiar with a resident to notice divergent behaviours, possible indicators of changing needs [11, 19, 27–30]. Mutual trust and understanding among nursing staff, other professionals, and relatives are crucial, as they rely on strong relations and awareness of each other’s knowledge, skills, and values. These elements are necessary to unite the diverse perspectives on the desired direction of care [31]. Stakeholders look at a resident trough their own, unique lenses; combining these perspectives will most likely represent the personal needs of the individual. In line with this, for further research it would be interesting to study the challenges as experienced by the relatives and the members of the multidisciplinary team.

As being familiar with each other benefits the provision of palliative care on many levels, organizations should focus on continuity of care and communication, especially when caring for people with serious cognitive impairment or severe dementia. Their quality of life depends to a great extent on the observations of others and communication between them. The importance of communication has already been emphasized by various other authors (e.g [7, 32–34]). The experiences of nursing staff in our study show that organizational structures can be a hindering factor in communication. Therefore, we advise organizations to invite nursing staff along with relatives and the multidisciplinary team to think about alternative structures to facilitate communication. Above that, more ad hoc consultations with the elderly care physician could be facilitated by the nursing home management.

The action research project (SigMa project) this focus group study was part of, aims at finding tools to support the process of recognizing and discussing changes in a resident’s condition. Tools might be useful to help nursing staff to make their experiential and relational knowledge explicit, or to put their “gut feeling” into words. This may make them feel more confident and may help in discussing their observations, and strengthen their position. Additionally, tools could be used in meetings with colleagues, relatives or the multidisciplinary team to facilitate discussing a resident’s condition [28] and achieve a consensus on the directions of care. It is important to discuss tools in the context of an organisation’s structure and culture to facilitate really meaningful use of these tools. Further research is needed to explore these options and to find out what types of tools nursing staff prefers.

### Methodological considerations

A strength of this study is the use of focus group discussions from 10 different nursing homes including nursing staff at different levels who all are important actors in daily practice regarding recognizing and discussing changes in a resident’s condition in the palliative phase. Another strength is the specific focus on *what exactly* makes signalling changes and needs of nursing home patients difficult for nursing staff. This is a first essential step in proving appropriate care to nursing home patients.

A limitation of this study is the recruitment of nursing home teams for another purpose than the focus group discussions only. We recruited them for an action research to improve palliative care. We therefore know that they were motivated to improve palliative care. As we asked managers to participate with teams that were open to change, the participating teams may be more reflective on their own actions. Therefore, our findings may provide a relatively positive account of the experiences and challenges of nursing home staff to identify needs.

## Conclusion

While supporting the observational skills of nursing staff is important, it is not enough to improve the quality of care for nursing home residents with palliative care needs. As nursing staff experiences challenges at different, interrelated levels, improving the process of recognizing and discussing changes in nursing home residents requires an integrated approach whereby the organization facilitates strengthening of the position of nursing staff. It is important that their observations become a valued and integrated and part of nursing home care.

### Electronic supplementary material

Below is the link to the electronic supplementary material.

Supplementary Material 1

## Data Availability

The datasets used and/or analysed during the current study are available from the corresponding author on reasonable request.
