# Supplementary material for: Challenges in recognizing and discussing changes in a resident’s condition in the palliative phase: focus group discussions with nursing staff working in nursing homes about their experiences
Source: BMC Palliat Care. 2024 Jun 10;23:144. doi: 10.1186/s12904-024-01479-3 (PMC11163817; doi:10.1186/s12904-024-01479-3)
Supplement: Supplementary file 1 — Supplementary Material 1 [file 12904_2024_1479_MOESM1_ESM.docx]

**Appendix 1: Topic list**

# Topic list focus group discussion SigMa:

**Attitude towards Recognizing and Discussing Changes**

- Initial associations with "recognizing and discussing changes in the palliative-terminal phase"
- Consequences of timely and accurate recognizing and discussing changes
  - Advantages
  - Disadvantages / Risks
- Involvement in the pre-project selection team for the SigMa project and planning

**Current Approach to Recognizing and Discussing Changes**

- Roles and responsibilities of FGI participants in recognizing and discussing changes
- Roles and responsibilities of other stakeholders
- How is the "feeling of unease" among caregivers addressed?
- How are residents and their loved ones involved?

**Evaluation of the Current Approach**

- What is going well?
  - What is the team proud of?
  - What positive feedback has the team received from residents, loved ones, or others?
- Examples of situations where recognizing and discussing changes went well
- Identified challenges
  - For example, knowledge, experience, resources, support from colleagues and management, communication
- Examples of situations where recognizing and discussing changes did not go well
- Support needs
  - Individual participants
  - Team level

**Use of Tools, General and Specific for Recognizing and Discussing Changes**

- Current use of tools
- Experienced / expected benefits of using tools
- Examples of situations where the use of tools added value
- Experienced / expected disadvantages or risks of using tools
- Examples of situations where the use of instruments provided no added value or had disadvantages
- Choice of tools
  - Involvement in the choice
  - Considerations for the choice

- How do tools align with the nursing home's practical context?

**Setting Objectives**

- What does the team stand for? How does recognizing and discussing changes fit in?
- What does the team want to work on?

**Conclusion**

- How did the team experience the FGD?
- Discussing the next steps.
